# Clinical impact of intra-aortic balloon pump during extracorporeal life support in patients with acute myocardial infarction complicated by cardiogenic shock

**DOI:** 10.1186/1471-2253-14-27

**Published:** 2014-04-14

**Authors:** Taek Kyu Park, Jeong Hoon Yang, Seung-Hyuk Choi, Young Bin Song, Joo-Yong Hahn, Jin-Ho Choi, Kiick Sung, Young Tak Lee, Hyeon-Cheol Gwon

**Affiliations:** 1 Department of Medicine, Division of Cardiology, Cardiac and Vascular Center, Samsung Medical Center, Sungkyunkwan University School of Medicine, 50 Irwon-dong, Gangnam-gu, Seoul 135-710, Republic of Korea; 2 Department of Critical Care Medicine, Samsung Medical Center, Sungkyunkwan University School of Medicine, Seoul, Republic of Korea; 3 Department of Thoracic and Cardiovascular Surgery, Cardiac and Vascular Center, Samsung Medical Center, Sungkyunkwan University School of Medicine, Seoul, Republic of Korea

**Keywords:** Myocardial infarction, Cardiogenic shock, Extracorporeal life support, Intra-aortic balloon pump

## Abstract

**Background:**

There is no available data on clinical outcome in patients with acute myocardial infarction (AMI) complicated by cardiogenic shock who are supported by an intra-aortic balloon pump (IABP) in combination with extracorporeal life support (ECLS).

**Methods:**

We analysed 96 consecutive patients with AMI and complicating cardiogenic shock who were assisted by an ECLS system between January 2004 and December 2011. The primary outcome was in-hospital mortality. The secondary outcomes were the success rate of weaning from ECLS and the lactate clearance for 48 hours (%).

**Results:**

A combination of IABP and ECLS was used in 41 (42.7%) patients. In-hospital mortality occurred for 51 patients (ECLS with IABP versus ECLS alone; 51.2% vs. 54.5%, *p* = 0.747). The success rate of weaning from ECLS was similar between the two groups (63.4% vs. 58.2%, *p* = 0.604). Complications such as ischemia of a lower extremity or bleeding at the ECLS insertion site (*p* = 0.521 and *p* = 0.667, respectively) did not increase when ECLS was combined with IABP. Among patients who survived for 24 hours after intervention, lactate clearance was not significantly different between patients who received ECLS alone and those who received ECLS with IABP (*p* = 0.918).

**Conclusions:**

The combined use of ECLS and IABP did not improve in-hospital survival in patients with AMI complicated by cardiogenic shock.

## Background

The mortality rate for acute myocardial infarction (AMI) complicated by cardiogenic shock appears to be unchanged at about 40-50% after the introduction of primary percutaneous coronary intervention (PCI) and an intra-aortic balloon pump (IABP) [1-3]. Accordingly, new mechanical-assist devices are being developed to maintain hemodynamic support in cardiogenic shock. Extracorporeal life support (ECLS) can be placed percutaneously and initiated quickly, making it helpful in emergencies [4]. ECLS, however, has an essential limitation: it is only able to partially unload the left ventricle (LV), and LV afterload may be high [5]. IABP may be an option to optimize hemodynamic status during ECLS and to reduce afterload and increase diastolic augmentation with an improvement in coronary perfusion [6]. There is limited data, however, about clinical outcomes of simultaneous IABP support and ECLS in AMI patients complicated by cardiogenic shock. Here, we report outcomes of this combination therapy.

## Methods

### Study population

**Figure 1:**
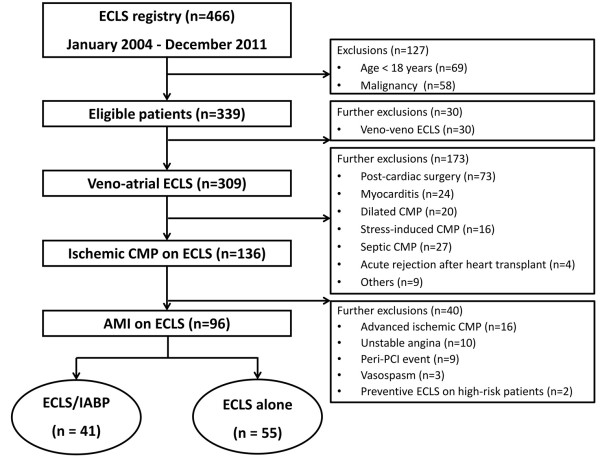
**Study population.** ECLS, extracorporeal life support; CMP, cardiomyopathy; AMI, acute myocardial infarction; IABP, intra-aortic balloon pump; PCI, percutaneous coronary intervention.

We retrospectively reviewed our registry of patients with ECLS between January 2004 and December 2011 (Figure 1). The study was approved by the Institutional Review Board of Samsung Medical Center. Patients were enrolled for the study if they presented with an AMI (with or without ST-elevation) complicated by cardiogenic shock or in-hospital arrest. A patient was diagnosed with cardiogenic shock if their systolic blood pressure was less than 90 mmHg for more than 30 minutes after correcting hypovolemia, hypoxemia, and acidosis with maximal medical treatment. An arrest was presumed to be of cardiac aetiology unless it was known or likely to have been caused by non-cardiac cause. We excluded patients with age >80 years, previous severe neurologic damage, those who previously signed a “do-not-resuscitate” order, and patients with irreversible organ failure who would not have received physiological benefits from this treatment.

### ECLS implantation

ECLS was performed when patients in cardiogenic shock were unresponsive to the administration of vasopressors after the correction of hypovolemia and hypoxemia or when arrest was prolonged or recurrent. The decision to implant ECLS, IABP, or both was determined by the experienced interventional cardiologists in charge. When patients with IABP deteriorated, we implanted ECLS therapy. All patients were expected to undergo early revascularization and to receive medical therapy in accordance with guidelines [7,8].

A Capiox Emergency Bypass System (Capiox EBS™; Terumo Inc., Tokyo, Japan) was used in all cases. This system is composed of a portable controller with a back-up battery, a disposable bypass circuit integrated with a heparin-coated membrane oxygenator, and a centrifugal pump. Device insertion was performed by percutaneous cannulation using the Seldinger technique. Surgical cannulation using the cut-down method was performed in difficult cases. Cannula sizes ranged from 14 to 21 French for the femoral artery and from 21 to 28 French for the femoral vein. In the event of distal limb ischemia after arterial cannulation, a catheter was inserted distal to the cannulation site for limb perfusion. In patients receiving combined ECLS and IABP, a 40-ml intra-aortic balloon was introduced via the femoral artery on the opposite side of the ECLS-inserted catheter. IABP support was initiated using 1:1 electrocardiographic triggering and was maintained until weaning from the ECLS.

### Patient management under ECLS

We previously reported detailed management [9] in which continuous unfractionated heparin was infused intravenously to maintain an activated clotting time between 180 and 220 sec. The initial revolutions per minute of the ECLS device and use of vasoactive drug were adjusted in order to achieve an ideal cardiac index greater than 2.2 L/min/body surface area (m^2^), central mixed venous saturation greater than 70%, and mean arterial pressure greater than 65 mmHg. Echocardiography was performed daily to monitor cardiac function. If the patient was hemodynamically stable and adequately oxygenated, they were considered for ECLS weaning when the flow rate was 1 L/min/m^2^ for four hours. Successful weaning was defined as disconnection of the patient from ECLS without reinsertion or death within 24 hours. After that time, weaning from IABP was achieved by reducing the trigger ratio. Termination of ECLS was considered, with the consent of the family, when there was intractable multi-organ failure or severe neurologic damage consistent with a vegetative state or brain death.

### Data collection and outcome variables

The primary outcome of the study was in-hospital mortality. To determine predictors of mortality, clinical data was obtained through medical record review. Detailed in-hospital data included age, gender, co-morbidities, and laboratory and procedural findings. Secondary outcomes included the success rate of weaning from ECLS and the lactate clearance for 48 hours. Lactate level was measured from arterial or venous blood samples. Lactate clearance for 48 hours (%) was defined according to the following formula: lactate clearance for 48 hours (%) = [(highest lactate level in the initial six hours – lowest lactate level in the first 24 to 48 hours)/highest lactate level in the initial six hours] × 100. Safety outcomes included limb ischemia requiring surgical or interventional therapy, bleeding requiring transfusion (decrease in haemoglobin greater than 2.0 g/dL), stroke (identified by the occurrence of new neurologic symptoms with the evidence of ischemia or haemorrhage on computed tomography), and sepsis with clinical signs of infection and growth of bacteria on blood culture.

### Statistical analysis

All values are presented as number with percentage for categorical variables and median with interquartile range for continuous variables. Comparisons between continuous variables were made using a *t*-test or Mann–Whitney *U* test, as appropriate. Categorical data were analysed using the Chi-square test or Fisher’s exact test, as appropriate. Event-free survival curves were estimated by the Kaplan-Meier method and compared using the log-rank test. Hazard rates were determined by survival analysis using the Cox proportional hazards model. In multivariable models, covariates included those with a p value < 0.2 and those that were clinically relevant. We selected the number of variables in each multivariable model in order to have approximately one variable for every ten patients so as to reduce overfitting. All tests were two-tailed, and *p* < 0.05 was considered statistically significant. SPSS version 20 (IBM, Armonk, New York, USA) was used for statistical analysis.

## Results

### Patient characteristics

Between January 2004 and December 2011, 96 patients presented with AMI complicated by cardiogenic shock and were enrolled in this study. IABP support was combined with ECLS in 41 patients (ECLS/IABP group), whereas 55 patients were supported by ECLS alone (ECLS alone group). Baseline characteristics and comparisons between the ECLS/IABP group and the ECLS alone group are shown in Table 1. Demographics and co-morbidities were similar between the two groups. There were no significant differences in the clinical course before initiation of mechanical-assist devices between the two groups, except for cardiopulmonary resuscitation (CPR). Sixty-one (63.5%) patients underwent CPR just before ECLS placement. There were fewer patients with cardiac arrest in the ECLS/IABP group than in the ECLS alone group [20/41 (48.8%) vs. 41/55 (74.5%), P = 0.009].

**Table 1:** Pre-ECLS characteristics

	**ECLS/IABP (n = 41)**	**ECLS alone (n = 55)**	**p value**
Age, years	66 (57–74)	64 (54–75)	0.591
Male gender	31 (75.6)	43 (78.2)	0.767
Body mass index, kg/m^2^	24.0 (22.4-25.5)	22.8 (21.1-25.2)	0.251
Diabetes mellitus	24 (58.5)	35 (63.6)	0.612
Hypertension	21 (51.2)	25 (45.6)	0.576
Dyslipidemia	5 (12.2)	9 (16.4)	0.567
Current smoker	17 (41.5)	22 (40.0)	0.885
Previous myocardial infarction	7 (17.1)	6 (10.9)	0.383
Previous PCI	7 (17.1)	9 (16.4)	0.926
Previous bypass surgery	3 (7.3)	1 (1.8)	0.310
Known peripheral arterial disease	4 (9.8)	3 (5.5)	0.456
Myocardial infarction			0.559
ST-elevation	26 (63.4)	38 (69.1)	
Non-ST-elevation	15 (36.6)	17 (30.9)	
Cardiopulmonary resuscitation	20 (48.8)	41 (74.5)	0.009
Systolic blood pressure, mmHg	78 (70–83)	80 (70–82)	0.868
Diastolic blood pressure, mmHg	49 (40–60)	50 (44–56)	0.906
Heart rate	113 (71–140)	92 (71–107)	0.285
Hemoglobin, g/dL	13.0 (10.6-14.8)	12.5 (10.3-14.4)	0.497
Creatinine, mg/dL	1.26 (1.07-1.78)	1.23 (0.97-1.75)	0.664
Lactate, initial, mmol/L	7.7 (2.7-13.1)	6.1 (3.4-9.5)	0.550
Left ventricular ejection fraction, %	30 (24–38)	40 (25–50)	0.109
Multi-vessel disease	33 (80.5)	36 (65.5)	0.105
Infarct-related artery			0.319
Left main	6 (14.6)	13 (23.6)	
Left anterior descending	19 (46.3)	29 (52.7)	
Left circumflex	7 (17.1)	4 (7.3)	
Right coronary	9 (22.0)	9 (16.4)	
Treatment strategies			0.056
PCI	33 (80.5)	45 (81.8)	
Bypass surgery	7 (17.1)	3 (5.5)	
Medical treatment	1 (2.4)	7 (12.7)	
Use of vasopressor/inotropic agent	41 (100)	54 (98.2)	1.000

Values are median (interquartile range) or n (%).

ECLS, extracorporeal life support; IABP, intra-aortic balloon pump; PCI, percutaneous coronary intervention.

PCI was used for revascularization in 78 (81.3%) patients. Ten (10.4%) patients underwent immediate bypass surgery or PCI with subsequent bypass surgery. Medical treatment was maintained in eight (8.3%) patients after coronary angiography. Six patients did not receive revascularization due to diffuse or distal coronary artery disease; two patients did not receive revascularization due to cannulation failure. Seven of these eight patients passed away.

### Clinical outcomes

**Figure 2:**
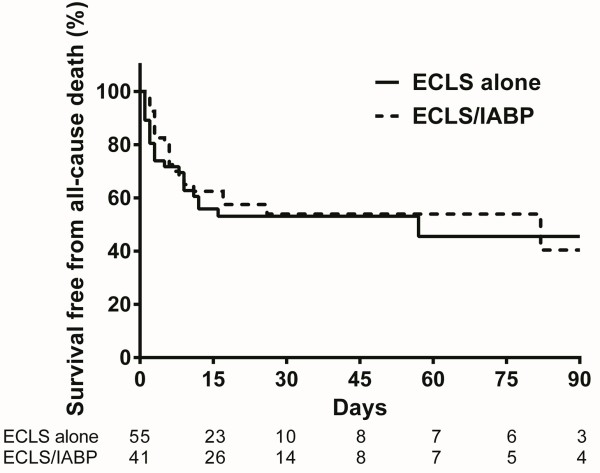
**A comparison of survival between the ECLS/IABP group (n = 41) and the ECLS alone group (n = 55).** ECLS, extracorporeal life support; IABP, intra-aortic balloon pump.

Observed clinical outcomes are shown in Table 2. The in-hospital mortality was similar among patients in the ECLS/IABP group and the ECLS alone group (51.2% and 54.5%, respectively; hazard ratio with additional IABP, 0.8; 95% confidence interval [CI] 0.457-1.399; P = 0.434). The cumulative rates of survival at 30 days in the ECLS/IABP group and the ECLS alone group were 53.9% and 53.1%, respectively (Figure 2). Additional IABP had no significant impact on in-hospital mortality in multivariate modelling after adjusting for age, sex, CPR, ST elevation myocardial infarction, heart rate, left ventricular ejection fraction, and treatment strategies (hazard ratio 0.972; 95% CI 0.506-1.866; P = 0.932).

**Table 2:** Clinical outcomes and complications

	**ECLS/IABP (n = 41)**	**ECLS alone (n = 55)**	**p value**
In-hospital death	21 (51.2)	30 (54.5)	0.747
ECLS weaning success	26 (63.4)	32 (58.2)	0.604
Lactate clearance for 48 hours, %*	65.2 (40.9-79.6)	65.3 (25.2-82.0)	0.918
Complications			
Limb ischemia	6 (15.0)	5 (9.4)	0.521
ECLS site bleeding	9 (22.5)	10 (18.9)	0.667
Gastrointestinal bleeding	9 (22.5)	6 (11.3)	0.147
Stroke	0	3 (7.5)	0.076
Sepsis	3 (7.5)	4 (7.5)	1.000

Values are median (interquartile range) or n (%).

ECLS, extracorporeal life support; IABP, intra-aortic balloon pump.

*Lactate clearance for 48 hours was available in 80 patients who survived at least 24 hours after intervention.

The success rates of weaning from ECLS were also similar between the two groups (63.4% vs. 58.2%, P = 0.604). Among patients who survived for 24 hours or more, there was no significant difference in lactate clearance for 48 hours between the ECLS/IABP group and the ECLS alone group. There were no significant differences between the ECLS/IABP group and the ECLS alone group with respect to limb ischemia, site bleeding, gastrointestinal bleeding, stroke, or sepsis.

## Discussion

We investigated whether additional IABP is associated with improved in-hospital survival in patients with AMI and complicating cardiogenic shock who underwent ECLS. The combined use of ECLS and IABP did not improve in-hospital survival or tissue hypoperfusion. The additional IABP insertion did not increase vascular complications or bleeding.

An IABP improves coronary and peripheral perfusion via diastolic balloon inflation and augments LV performance via systolic balloon deflation, with an acute decrease in afterload [10]. The effects of IABP during ECLS, however, have not been evaluated sufficiently. An experimental study suggested that IABP-induced pulsatility improves coronary bypass graft flows during nonpulsatile ECLS [11]. Another study proposed that adjunctive IABP improves the myocardial oxygen supply–demand balance in peripheral extracorporeal life support [12]. These benefits of IABP might increase coronary flow during diastole and compensate for limitations of ECLS, such as myocardial oxygen consumption associated with increased LV afterload. Consequently, the concomitant use of IABP might improve in-hospital survival during ECLS management. In our study of patients with AMI and complicating cardiogenic shock, however, combined IABP support during ECLS did not reduce in-hospital mortality. A recent study in Korea also showed that the concomitant use of IABP did not reduce hospital mortality despite an improved success rate in weaning from ECLS [13]. The most likely explanation about different successful weaning rate involves patient selection. Our patients had a higher incidence of cardiac arrest compared to that in the previous study (63.5% vs. 31.6%). A large proportion of patients in their study (46.6%) were enrolled for low cardiac output after cardiac surgery, and it is therefore difficult to compare their results directly to ours.

To the best of our knowledge, there have been no well-designed trials to explain why there are no significant differences between mortality in the two groups. We considered the possibility that coronary blood flow might not increase enough to cause haemodynamic changes after using IABP under continuous flow from ECLS into the human circulating system. There were no significant differences in haemodynamic variables, such as cardiac output, between the patients supported by IABP and the patients treated with medical therapy in a recent randomized trial, although that study was conducted without ECLS management [14]. Furthermore, the IABP balloon may cause intermittent aortic occlusion, which may diminish the blood flow from ECLS to the aortic root and coronary arteries. An experimental study demonstrated that, in the setting of cardiac arrest, the addition of IABP to ECLS might worsen coronary blood flow [15].

Because lactate clearance early in hospital care may indicate a resolution of global tissue hypoxia [16], we compared the lactate clearance for 48 hours between the ECLS/IABP group and the ECLS alone group to evaluate the effectiveness of IABP for aiding recovery from tissue hypoperfusion during cardiogenic shock. Lactate clearance for 48 hours, however, was similar regardless of the combined use of IABP. This finding suggests that additional IABP support is unlikely to be useful in the early recovery from severe cardiogenic shock.

Our study had several limitations. First, it was a retrospective, observational study; therefore, confounding factors may have significantly affected our results. Hidden biases for the initiation of mechanical-assist devices might exist because the attending physician in charge decided whether to use ECLS alone or with IABP. Furthermore, the ECLS/IABP group included patients who were initially supported by IABP alone and later also received ECLS. Our hypothesis was that the effect of LV unloading by IABP during ECLS might improve survival. Therefore, patients were placed in the ECLS/IABP group if they were supported by the IABP during ECLS, regardless of the implantation order. Second, our registry did not include pulmonary capillary wedge pressure measured by pulmonary arterial catheter, LV size, or E/e’ measured by echocardiography. This haemodynamic information might support the hypothesis of IABP’s effect on LV unloading. Third, performance of CPR, which has been reported as an important predictor of mortality in patients with severe cardiogenic shock [17,18], was significantly different between the two groups. In addition, a larger number of patients treated with only medical therapy without revascularization were included in the ECLS alone group, and a larger number of patients who had undergone bypass surgery were included in the ECLS/IABP group. Efforts were made to compensate for these differences in the multivariable analysis, but we may not have been able to overcome the differences. Fourth, our data included a large number of patients who underwent CPR and thus showed a higher mortality than in another recent randomized trial and a registry of cardiogenic shock [3,19]. Even if there are minor benefits of IABP support, additional effects might not be evident in patients with very severe cardiogenic shock. As there have been no other clinical studies regarding the use of IABP during ECLS, our study provides the first information about modalities of AMI shock treatment. A large registry study or randomized trial, however, is needed for a more complete assessment of the role of IABP during ECLS.

## Conclusions

Additional use of IABP during ECLS did not improve in-hospital survival or tissue perfusion in patients with AMI complicated by cardiogenic shock.

## Abbreviations

AMI: Acute myocardial infarction; PCI: Percutaneous coronary intervention; IABP: Intra-aortic balloon pump; ECLS: Extra-corporeal life support; LV: Left ventricle; CPR: Cardiopulmonary resuscitation.

## Competing interests

The authors declare that they have no competing interests.

## Authors’ contributions

All authors contributed to the study design, acquisition of data, or analysis and interpretation of data and have been involved in drafting the manuscript. JHY, S-HC, YBS, J-YH, J-HC, KS, YTL and H-CG participated in the enrolment of patients, performed the procedures, and contributed to clinical follow-up. TKP, JHY, S-HC, YBS, J-YH, J-HC, KS, YTL and H-CG participated in data collection. TKP, JHY, and S-HC participated in the data analysis. TKP, JHY, S-HC, YBS, J-YH, J-HC, KS, YTL and H-CG contributed to data interpretation. TKP, JHY and S-HC contributed to writing of the manuscript. All authors gave final approval of the manuscript for publication.

## Pre-publication history

The pre-publication history for this paper can be accessed here:

http://www.biomedcentral.com/1471-2253/14/27/prepub
